# Medical Items

**Published:** 1910-08

**Authors:** 


### MEDICAL ITEMS

Battle & Co., of St. Louis, have just issued No. 13 of their series of charts on dislocations. This series forms a most valuable and interesting addition to any physician's library. They will be sent free of charge on application, and back numbers will also be supplied. If you have missed any of these numbers, better write Battle & Co., for them before the supply is exhausted.

---

### THE DOSE OF CODEINE.

Fraenkel (*Munch. Med. Woch.*) claims that coedine must be given in larger doses than is generally used in order that the full effect may be obtained, as codeine is from ten to twenty times less powerful than morphine. The proper dose should be ewo-thirds or three-fourths grain, and this amount may be given three or four times a day without any evidence of habit formation. The single maximum dose permissible is one and one-half grains and maximum daily dose is four and one-half grains. For children the daily dose may be as follows:

| | |
|---|---|
| 4 years of age | 1-6 grain |
| 6 years of age | 1-3 grain. |
| 8 years of age | 2-3 grain. |
| 12 years of age | 1¼ garins. |

—*Meyer Brothers Druggist,* July, 1910.

### DOCTOR REMEMBER—

*Dioviburnia* is the most efficient uterine tonic, alternative, anti-spasmodic and anodyne, indicated in anemia, parturition, leucorrhea, menorrhagia, subinvolution, dysmenorrhea, miscarriage, vescial tenesmus, diarrhea, dysenter, chlorosis, ovaritis, cholera morbus, climacteric diseases, endometritis, uterine engorgement, metrorrhagia, prolapsus uteri, threatened abotrion, puerperal convulsions, vomiting of pregnancy, relaxed condition of the utereus.

*Neurosine* is the standard neurotic, anodyne and hypnotic. Contains no opium, morphine, chloral or other deleterious drugs. Indicated in all neuroses, migraine, hysteria, spermatorrhea, uterine congestion, shingles, opium habit, chorea, neuralgia, asthma, neurasthenia, herpes delirium tremens, whooping cough, sea sickness, and all reflex and convulsive neuroses.

